# Efficacy of a Digital Educational Intervention for Patients With Type 2 Diabetes Mellitus: Multicenter, Randomized, Prospective, 6-Month Follow-Up Study

**DOI:** 10.2196/60758

**Published:** 2025-04-10

**Authors:** Irene Caballero Mateos, Cristóbal Morales Portillo, María Lainez López, Ángel Vilches-Arenas

**Affiliations:** 1 Endocrinology and Nutrition Department Vithas Hospital Sevilla Spain; 2 Endocrinology and Nutrition Department Juan Ramón Jiménez University Hospital Huelva Spain; 3 Department of Preventive Medicine and Public Health Faculty of Medicine University of Seville Sevilla Spain

**Keywords:** body composition, type 2 diabetes mellitus, digital, metabolic control, social networks, satisfaction, telemedicine

## Abstract

**Background:**

Adherence to therapies and metabolic control among patients with type 2 diabetes mellitus (T2DM) remain challenging. The use of new technologies, such as telemedicine, digitalized systems, and social networks, could improve self-management and disease control.

**Objective:**

We evaluated the efficacy of a digital educational intervention for patients with T2DM, expressed as changes in glycated hemoglobin (HbA_1c_) and body composition and evaluation of the response using validated questionnaires of satisfaction with health care professionals (Instrument for Evaluation of the Experience of Chronic Patients), Diabetes Knowledge Scale (ECODI), and adherence to treatment over 6 months of follow-up (Morisky, Green, Levine Medication Assessment Questionnaire).

**Methods:**

This multicenter, randomized, prospective study included adults with T2DM with poor metabolic control who started treatment with glucagon-like peptide-1 receptor agonists. Patients were randomized to digital intervention or usual care. The intervention group received education through social networks and digital tools in a structured program of healthy lifestyle changes. This was provided by a “Digital Coach” for weekly and on-demand advice and individualized support. Baseline and follow-up demographic, clinical parameter, adherence, and quality of life data were collected.

**Results:**

We included 85 patients (control: n=41; intervention: n=44). Both groups were matched regarding demographics, physical examination, insulin, and biochemical parameters. We observed a reduction in body weight (intervention: –8.7, SD 6.1 kg vs control: –4.9, SD 5.0 kg; *t*_83_=–3.13; *P*=.002), BMI (intervention: –3.0, SD 2.1 kg/m^2^ vs control: –1.8, SD 1.8 kg/m^2^; *t*_83_=–2.82; *P*=.006), and fast mass in both groups but greater in the intervention group. There were greater reductions in fasting plasma glucose (intervention: 122.6, SD 81.5 mg/dL vs control: 70.5, SD 72.9 mg/dL; *t*_83_=3.10; *P*=.004) and HbA_1c_ (intervention: 3.7%, SD 1.9% vs control: 2.6%, SD 2.1%; *t*_83_=2.54; *P*=.006) in the intervention group. Although there was no significant change in the Spanish version of the Diabetes Quality of Life Questionnaire (EsDQOL) satisfaction score in the control group after 6 months of follow-up (0.7, SD 19.8), there was a marked reduction in EsDQOL satisfaction score in the intervention group (–13.7, SD 23.1; *t*_83_=–3.08; *P*=.02). According to the ECODI scale, knowledge about diabetes increased more in the intervention group (intervention: 0.3, SD 1.8 vs control: 1.5, SD 1.5; *t*_83_=–3.33; *P*=.001). Although the medication adherence score worsened in the control group after 6 months, it significantly improved with the intervention (control: –8% vs intervention: 13.8%; χ^2^_1_=0.35; *P*=.01). Patients’ health care experiences improved with the intervention but not with the control.

**Conclusions:**

The digital educational intervention was effective at improving glycemic control, body composition, adherence, and patient satisfaction compared with usual care in patients with T2DM. The implementation of digital tools and social media could highly improve the multidisciplinary approach to the management of this population.

**Trial Registration:**

ClinicalTrials.gov NCT06850129; https://clinicaltrials.gov/study/NCT06850129

## Introduction

Diabetes mellitus (DM) is one of the fastest growing global health emergencies of this century [1]. Type 2 DM (T2DM) accounts for over 90% of diabetes worldwide and can be prevented or delayed [2]. In Spain, the overall prevalence of DM is 14%, of which about one-half are underdiagnosed [3].

Attaining glycated hemoglobin (HbA_1c_) targets is mandatory to reduce the DM-related complications that are associated with considerable health care resource utilization and costs [4,5]. Although a number of new antidiabetic drugs have emerged recently to improve metabolic control in patients with T2DM, approximately only 50% to 60% of patients achieve recommended goals, and this proportion has not improved in the last decade [6,7]. Therefore, many of these patients require injectable medications, including glucagon-like peptide-1 (GLP-1) receptor agonists and insulin to achieve HbA_1c_ goals [8].

Poor adherence to pharmacological and nonpharmacological approaches, as well as therapeutic inertia, are important determinants of low metabolic control [9]. In this context, improving patient self-care and empowerment could have a positive impact [10,11]. The use of new technologies, such as telemedicine, digitized systems, and social networks, could enhance the management and control of T2DM [12-20]. Unfortunately, there are still many barriers to the adoption of digital health tools in clinical practice [19]. In addition, the information currently available about the impact of the application of eHealth solutions or social networks for patients with T2DM remains insufficient in Spain [20,21].

The main objective of this study was to analyze the effectiveness, expressed as a change in HbA_1c_, of an educational intervention delivered through digitized systems and the use of social networks within 6 months in a representative sample of patients with T2DM. In addition, variations in basal values of body weight and glucose parameters obtained after the educational intervention compared with no intervention were also analyzed.

## Methods

### Study Design

This was a multicenter, randomized, prospective, interventional study that included adults with T2DM who started treatment with GLP-1 receptor agonists, had poor metabolic control (HbA_1c_ >7%), were able to use a smartphone-based home digital tool, and had access to social networks and webinars focused on diabetes education. In addition, the patients had to be on stable doses of oral antidiabetics for at least the previous 90 days. In contrast, patients with a lack of motivation or who were unable to use social networks, were unable to read or write in Spanish, had a previous history of psychiatric disorder or serious organic disease, were using systemic corticosteroids (>10 days in treatment), or were pregnant were excluded. Outpatients were recruited during a 1-year period from the Endocrinology Departments of the Virgen Macarena Hospital (Seville) and Juan Ramón Jiménez Hospital (Huelva).

**Figure 1:**
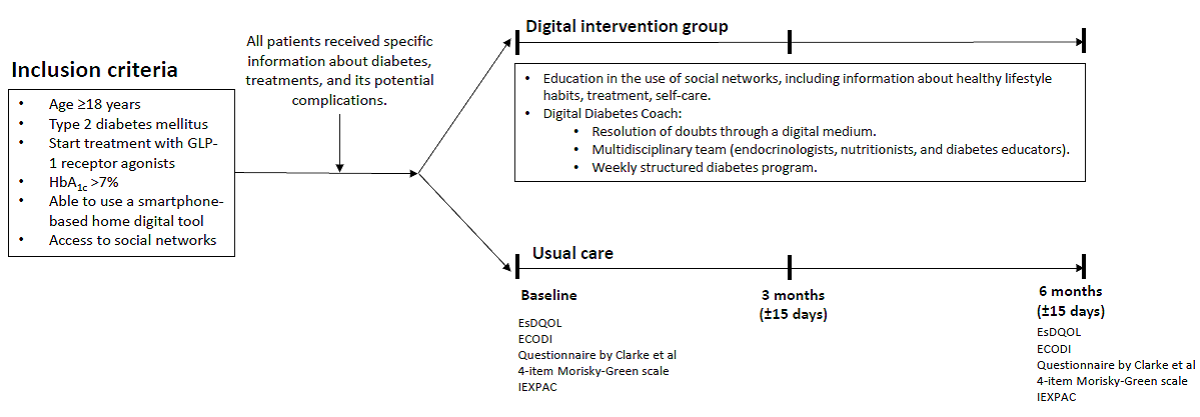
Study design. ECODI: Diabetes Knowledge Scale; EsDQOL: Spanish version of the Diabetes Quality of Life Questionnaire; GLP-1: glucagon-like peptide-1; HbA_1c_: glycated hemoglobin; IEXPAC: Instrument for Evaluation of the Experience of Chronic Patients.

The design of the study is summarized in Figure 1.

All patients received specific information about DM, its treatments, and potential complications. Patients were then randomized to the digital intervention group or usual care. Those patients randomized to the intervention group were educated on the use of different social networks with access to information related to healthy lifestyle habits, dietary support, optimization of physical activity, insulin titration, therapeutic targets, and self-care based on validated material from scientific societies and endorsed in diabetes education. Patients also had the valuable resource of a Digital Diabetes Coach: an online expert specializing in therapeutic education. This virtual guide provided patients with the flexibility to interact in both unidirectional and bidirectional ways, using methods such as chat boxes, audio messages, videos, and more. Through this digital medium, patients could seek guidance and have their questions resolved. The Digital Diabetes Coach operated under the supervision of a multidisciplinary team, endocrinologists, nutritionists, and certified diabetes educators. This team ensured that patients received comprehensive support and guidance tailored to their individual needs. Patient interactions were facilitated through a variety of digital platforms, which were selected based on the patient’s preferences and in consultation with their Digital Diabetes Coach. These interactions aimed to provide essential support and motivation for patients. The communication channels included email, Skype, WhatsApp, telephone calls, and video conferencing, allowing for flexibility and convenience in connecting with patients. The main communication channels used in this study included telephone calls, WhatsApp, email, and Skype video calls. Among them, WhatsApp was the preferred application by patients to ask questions and share information with professionals, regardless of their educational and socioeconomic level. Patients with higher educational levels also felt comfortable communicating through videoconferencing, while this medium was not the most desired by patients with a lower educational level or those from rural areas. In addition, real-time or recorded webinars through Instagram were also used. Furthermore, a comprehensive diabetes education program was introduced on a weekly basis, with 30-minute to 45-minute sessions. This program covered a wide spectrum of critical topics, including proper injection techniques, dosage adjustments for medications, strategies for managing adverse events, personalized recommendations for physical activity, guidance on blood glucose profiling and interpretation, strategies for handling hypoglycemia and hyperglycemia, portion-controlled dietary plans, and insights into potential health conditions associated with poor macrovascular and microvascular control. The educational materials used for these sessions were sourced from scientific societies, external companies endorsed by these societies, and materials developed by our center. To ensure effective communication and timely assistance, a 2-way communication system was established. Patients were encouraged to seek answers to their questions and access information on demand through messenger apps and other digital platforms. This approach allowed for a responsive and patient-centered approach to addressing individual needs and concerns. During the different interventions, patients were encouraged to send data such as weight, capillary blood glucose, and step count through activation of pedometers on their mobile phones.

In the control group, visits to the specialists (ie, endocrinologist) occurred every 3 months and included diabetes education intervention by the nursing staff (ie, use of medication, adherence to treatment, and basic guidelines for the management of their diabetes); each consultation lasted for about 30 minutes.

At baseline, demographic data, educational level, employment status, physical examination data, diabetes treatment (total insulin dose), and analytical data within the 12 weeks before inclusion were collected. Data were obtained from each electronic clinical history, and glucose levels were collected from the Libreview system. During the intermediate visit (3 months ± 15 days from baseline), we recorded information from a physical examination, the number of scans, medication adherence, verification that the patient adequately understood the material delivered and made use of social networks, changes in treatment, regular diabetes education, and adverse events since the last visit. As this was a routine visit, the education activities conducted by the staff (nursing and endocrine specialist) were homogeneous in both groups. At study end (6 months ± 15 days from baseline), we recorded information from a physical examination, number of scans, medication adherence, verification that the patient adequately understood the material delivered and made use of social networks, changes in treatment, regular diabetes education, and adverse events since the last visit. For changes in treatment, the total dose of insulin, rather than maximum or minimum doses of insulin, was considered. We also analyzed changes in body composition throughout the study, including body fat mass, visceral fat area, fat-free mass, and skeletal muscle mass using segmental analysis of multifrequency bioelectrical impedance (Inbody 770, Inbody Co Ltd) [22,23].

Additionally, at baseline and study end, different validated questionnaires were completed by the patients.

Quality of life was evaluated with the Spanish version of the Diabetes Quality of Life Questionnaire (EsDQOL) [24,25]. This questionnaire has 4 sections: satisfaction (15 questions, each with a score that ranges from 1 [very satisfied] to 5 [not at all satisfied]; 15 points implies great satisfaction), impact (17 questions, each with a score that ranges from 1 [never] to 5 [always]; 17 points indicates that DM has little impact on daily life), social or vocational concerns (7 questions, each with a score that ranges from 1 [never] to 5 [always]; 7 points indicates that DM causes little worry on a daily basis), and DM-related concerns (4 questions, each with a score that ranges from 1 [never] to 5 [always]; 4 points indicates that diabetes causes little worry on a daily basis).

The knowledge that patients with DM had about diabetes was evaluated using the Diabetes Knowledge Scale (ECODI) [26]. This scale has 25 items with 4 possible answers.

The 8 items of the questionnaire by Clarke et al [27] were used to evaluate the perception of hypoglycemia. This scale has a total score ranging from 0 to 7. Higher scores indicate diminished awareness.

The 4-item Morisky, Green, Levine Medication Assessment Questionnaire [28] was used to evaluate medication adherence. This scale has a total score of 0 to 4 points. A score of 4 denotes high adherence.

Patients’ health care experiences were assessed with the Instrument for Evaluation of the Experience of Chronic Patients (IEXPAC) questionnaire [29]. This is a self-administered, 12-item, multiple-choice questionnaire. Items 1 to 10 describe the patient’s experience in the last 6 months in 3 domains (productive interactions, new model of the patient’s relationship or interaction with the health care system, and patient’s self-care). The last 2 questions only apply to patients who have been hospitalized and are not counted in the total. As a result, the overall score ranges from 0 (worst experience) to 10 (best experience).

### Statistical Analysis

For the descriptive analysis, qualitative variables were defined using their absolute (n) and relative (%) frequencies, and quantitative variables were defined using measures of centralization (mean) and dispersion (SD). Categorical variables were compared with the chi-square or the Fisher exact test, when appropriate. To compare 2 means between groups (at baseline and changes during the study), parametric (Student *t* test) and nonparametric (Mann-Whitney *U* test) tests were used, as required. Hypothesis tests were 2-tailed in all comparisons, with a significance level of <.05. Statistical analyses were performed using SPSS v17.0 (IBM Corp).

### Ethical Considerations

This study was approved by the Clinical Research Ethics Committee of the Virgen Macarena – Virgen del Rocío University Hospital Center in Seville, Spain on May 18, 2020. The study was carried out in accordance with the requirements expressed in the Declaration of Helsinki and Good Epidemiological Practices, as well as the current legislation of Spain (Organic Law 3/2018, of December 5, on the Protection of Personal Data and guarantee of digital rights). Only the investigators and technical staff participating in the study had access to the patients' data in order to preserve their confidentiality. Every patient who participated in the study signed the corresponding informed consent form after receiving a full explanation of the conditions for the study and procedures to be performed. There was no financial compensation for patients or researchers nor any source of funding that could lead to a conflict of interest for the study.

## Results

**Figure 2:**
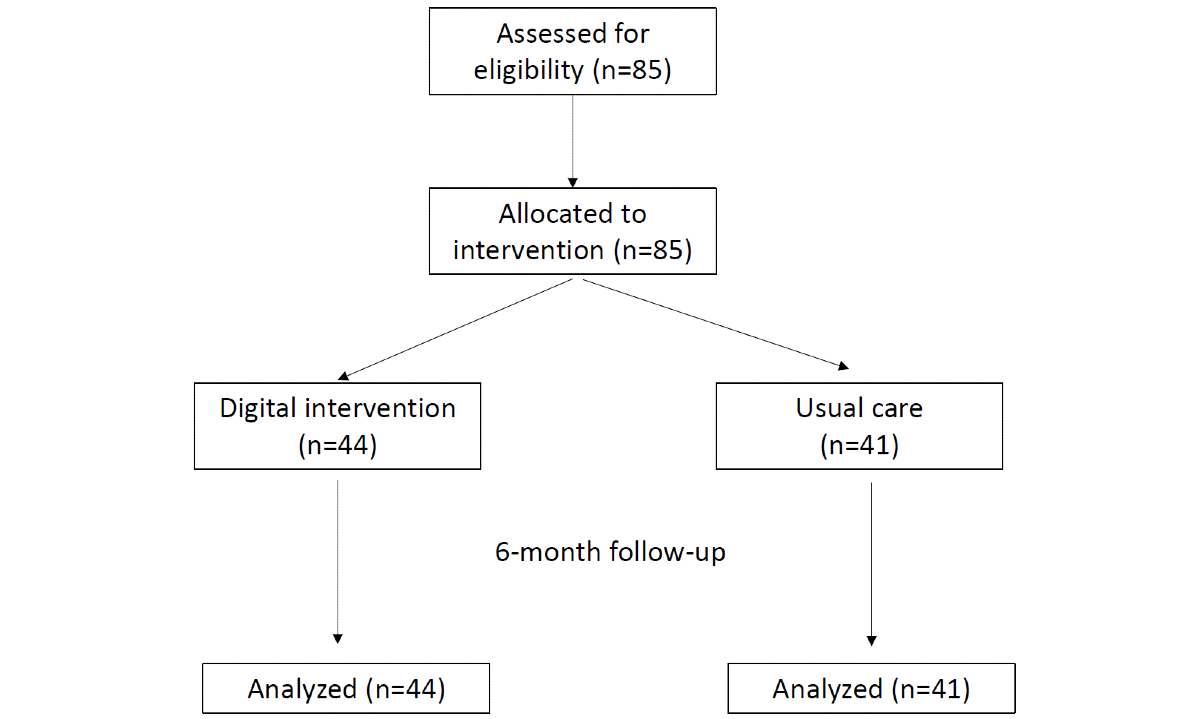
Study flowchart.

A total of 85 patients (41 in the control group and 44 in the intervention group) were included in the study (Figure 2).

The baseline clinical characteristics of the study population are presented in Table 1. In general, the 2 groups (control group vs intervention group) were well matched regarding demographic data, physical examination, insulin treatment, biochemical parameters, and the questionnaire scores. However, there were significant differences in some variables, such as body weight (control: 98.3, SD 14.3 kg vs intervention: 105.4, SD 16.4 kg; *P*=.04) and some parameters of body composition including extracellular water (control: 15.1, SD 2.8 L vs intervention: 17.8, SD 2.9 L; *P*<.001), body fat mass (control: 44.9, SD 11.4 kg vs intervention: 52.2, SD 18.8 kg; *P*=.03), fat-free mass (control: 53.4, SD 10.1 kg vs intervention: 62.1, SD 9.1 kg; *P*<.001), skeletal muscle mass (control: 29.6, SD 6.0 kg vs intervention: 34.6, SD 5.3 kg; *P*<.001), and skeletal muscle index (control: 8.1, SD 1.0 vs intervention: 8.9, SD 1.3; *P*<.001; Table 1).

**Table 1:** Baseline clinical characteristics based on study group.

Characteristics				Control group (n=41)		Intervention group (n=44)		*P* value
**Biodemographic data**								
	Age (years), mean (SD)			54.2 (12.9)		53.0 (10.9)		.65
	Sex (men), n (%)			20 (49)		28 (64)		.17
	Duration of diabetes (years), mean (SD)			9.1 (3.5)		8.9 (3.8)		.80
	**Educational level, n (%)**							.04
		No education	1 (2)		0 (0)			
		Primary	2 (5)		4 (9)			
		Secondary	24 (59)		21 (48)			
		University	2 (5)		14 (32)			
		Not available	12 (29)		5 (11)			
	**Employment status, n (%)**							.06
		Active	13 (32)		23 (52)			
		Retired	11 (27)		6 (14)			
		Unemployed	6 (15)		10 (23)			
		Not available	11 (27)		5 (11)			
**Physical examination, mean (SD)**								
	Weight (kg)			98.3 (14.3)		105.4 (16.4)		.04
	BMI (kg/m^2^)			36.8 (5.2)		37.1 (5.6)		.80
	SBP^a^ (mm Hg)			138.0 (18.4)		132.9 (13.7)		.15
	DBP^b^ (mm Hg)			78.5 (9.6)		78.8 (9.6)		.89
	HR^c^ (bpm)			93.9 (9.6)		84.9 (13.6)		.001
**Body composition, mean (SD)**								
	Extracellular water (L)			15.1 (2.8)		17.8 (2.9)		<.001
	Extracellular water to total body water ratio			0.39 (0.06)		0.39 (0.009)		.99
	Body fat mass (kg)			44.9 (11.4)		52.2 (18.8)		.03
	Visceral fat area (cm^2^)			217.2 (43.4)		225.0 (61.4)		.50
	Fat-free mass (kg)			53.4 (10.1)		62.1 (9.1)		<.001
	Skeletal muscle mass (kg)			29.6 (6.0)		34.6 (5.3)		<.001
	Skeletal muscle index			8.1 (1.0)		8.9 (1.3)		.002
	Phase angle			5.3 (0.7)		5.2 (0.7)		.51
**Insulin treatment, mean (SD)**								
	Basal insulin (UI)			25.3 (18.8)		26.7 (19.8)		.74
	Rapid-acting insulin (UI)			24.7 (13.8)		30.1 (21.5)		.17
	Total insulin dose (UI)			32.5 (23.1)		38.6 (36.8)		.36
**Biochemical parameters, mean (SD)**								
	Fasting glucose (mg/dL)			199.4 (66.3)		231.0 (80.5)		.051
	HbA_1c_^d^ (%)			9.5 (1.8)		9.9 (1.7)		.30
	TC^e^ (mg/dL)			201.4 (57.9)		186.1 (43.4)		.18
	LDL-c^f^ (mg/dL)			100.1 (58.8)		94.1 (37.0)		.58
	HDL-c^g^ (mg/dL)			47.2 (21.4)		43.0 (19.1)		.34
	TG^h^ (mg/dL)			230.5 (140.6)		227.2 (161.7)		.92
	eGFR^i^ (mL/min/1.72 m^2^)			95.4 (23.8)		91.8 (19.6)		.45
**Questionnaires, mean (SD)**								
	EsDQOL^j^ satisfaction			38.2 (12.5)		44.2 (19.2)		.09
	EsDQOL impact			33.8 (10.4)		31.5 (10.9)		.32
	EsDQOL social/vocational concerns			11.5 (6.7)		8.8 (4.8)		.04
	EsDQOL diabetes-related concerns			12.3 (4.5)		10.6 (5.0)		.10
	ECODI^k^			8.0 (2.0)		8.2 (1.7)		.62
	Morisky-Green^l^			0.5 (0.5)		0.5 (0.5)		.99
	IEXPAC_1-11_^m^			9.0 (1.5)		8.5 (1.4)		.12
	IEXPAC_12_			8.5 (2.1)		6.9 (2.9)		.004
	Questionnaire by Clarke et al [27]			0.5 (0.5)		0.4 (0.5)		.36

^a^SBP: systolic blood pressure.

^b^DBP: diastolic blood pressure.

^c^HR: heart rate.

^d^HbA_1c_: glycated hemoglobin.

^e^TC: total cholesterol.

^f^LDL-c: LDL cholesterol.

^g^HDL-c: HDL cholesterol.

^h^TG: triglycerides.

^i^eGFR: estimated glomerular filtration rate.

^j^EsDQOL: Spanish version of the Diabetes Quality of Life Questionnaire.

^k^ECODI: Diabetes Knowledge Scale.

^l^MGL MAQ: Morisky, Green, Levine Medication Assessment Questionnaire.

^m^IEXPAC: Instrument for Evaluation of the Experience of Chronic Patients.

The evolution of physical examination parameters and body composition after 6 months of follow-up are presented in Table 2 and Multimedia Appendix 1. Although there was a reduction in body weight and BMI in both groups, this decrease was greater in the intervention group: control –4.9 (SD 5.0) kg vs intervention –8.7 (SD 6.1) kg (*P*=.002) and control: –1.8 (SD 1.8) kg/m^2^ vs intervention: –3.0 (SD 2.1) kg/m^2^ (*P*=.006), respectively. Compared with the control group, there was a higher reduction with the intervention in extracellular water (control: –0.06, SD 0.5 L vs intervention: –0.5, SD 0.9 L; *P*=.03), body fat mass (control: –4.4, SD 3.7 kg vs intervention: –8.7, SD 5.2 kg; *P*=.002), fat-free mass (control: –0.34, SD 2.0 kg vs intervention: –2.8, SD 4.6 kg; *P*=.02), and skeletal muscle index (control: –0.009, SD 0.4 vs intervention: 0.3, SD 0.4; *P*=.005).

**Table 2:** Evolution of physical examination parameters and body composition after 6 months of follow-up based on study group.

Characteristics		Control group (n=41)					Intervention group (n=44)					
		Baseline	Study end	Difference	*P* value^a^	Baseline		Study end	Difference	*P* value^a^		
**Physical examination, mean (SD)**												
	Weight (kg)	98.3 (14.3)	93.4 (15.3)	–4.9 (5.0)	.002	105.4 (16.4)		96.7 (15.6)	–8.7 (6.1)	.002		
	BMI (kg/m^2^)	36.8 (5.2)	34.9 (5.6)	–1.8 (1.8)	.006	37.1 (5.6)		34.0 (5.5)	–3.0 (2.1)	.006		
	SBP^b^ (mm Hg)	138.0 (18.4)	140.3 (29.8)	–2.3 (0)	.39	132.9 (13.7)		126.6 (18.4)	–6.3 (0)	.32		
	DBP^c^ (mm Hg)	78.5 (9.6)	73.3 (9.3)	–5.2 (0)	.13	78.8 (9.6)		80.7 (10.6)	–1.9 (0)	.03		
	HR^d^ (bpm)	93.9 (9.6)	90.0 (7.2)	–3.9 (0)	.95	84.9 (13.6)		77.8 (9.6)	–7.1 (0)	.64		
**Body composition, mean (SD)**												
	Extracellular water (L)	15.1 (2.8)	15.0 (3.0)	–0.06 (0.5)	.03	17.8 (2.9)		17.2 (2.7)	–0.5 (0.9)	.03		
	Extracellular water to total body water ratio	0.39 (0.06)	0.39 (0.008)	0.0007 (0.005)	.92	0.39 (0.009)		0.39 (0.008)	0.006 (0.005)	.92		
	Body fat mass (kg)	44.9 (11.4)	40.5 (12.2)	–4.4 (3.7)	.002	52.2 (18.8)		43.5 (19.2)	–8.7 (5.2)	.002		
	Visceral fat area (cm^2^)	217.2 (43.4)	43.4 (197.9)	–19.3 (24.1)	.06	225.0 (61.4)		190.6 (67.0)	–34.5 (30.0)	.09		
	Fat-free mass (kg)	53.4 (10.1)	53.1 (11.2)	–0.34 (2.0)	.02	62.1 (9.1)		59.3 (9.9)	–2.8 (4.6)	.02		
	Skeletal muscle mass (kg)	29.6 (6.0)	29.4 (6.8)	–0.22 (1.3)	.051	34.6 (5.3)		33.5 (5.2)	–1.1 (1.7)	.051		
	Skeletal muscle index	8.1 (1.0)	8.1 (1.1)	–0.009 (0.4)	.005	8.9 (1.3)		8.6 (1.2)	–0.3 (0.4)	.005		
	Phase angle	5.3 (0.7)	5.2 (0.8)	–0.056 (0.3)	.62	5.2 (0.7)		5.1 (0.7)	–0.1 (0.4)	.47		

^a^Difference from baseline.

^b^SBP: systolic blood pressure.

^c^DBP: diastolic blood pressure.

^d^HR: heart rate.

After 6 months of follow-up, there was a significant decrease in insulin use in both groups, with a trend toward a greater decrease in total insulin dose in the intervention group, but this difference was not statistically significant (control: –2.7, SD 18.1 UI vs intervention: –11.5, SD 20.6 UI; *P*=.09). Regarding the biochemical parameters, there were significant reductions from baseline values in fasting plasma glucose and HbA_1c_ levels in both groups, but these decreases were greater in the intervention group (control: –70.5, SD 72.9 mg/dL vs intervention: –122.6, SD 81.5 mg/dL; *P*=.004 and control: –2.6%, SD 2.1% vs intervention: –3.7%, SD 1.9%; *P*=.006, respectively). Similar improvements in lipid profile were observed in both groups (Table 3 and Multimedia Appendix 2).

**Table 3:** Evolution of insulin treatment and biochemical parameters after 6 months of follow-up by study group.

Characteristics			Control group (n=41)									Intervention group (n=44)						
			Baseline		Study end		Difference		*P* value^a^		Baseline			Study end		Difference		*P* value^a^
**Insulin treatment, mean (SD)**																		
	Basal insulin (UI)	25.3 (18.8)		23.7 (16.6)		–1.0 (14.3)		.21		26.7 (19.8)			20.4 (24.3)		–5.9 (13.1)		.04	
	Rapid-acting insulin (UI)	24.7 (13.8)		7.5 (17.2)		–10.3 (28.7)		.56		30.1 (21.5)			6.8 (16.8)		–15.5 (11.7)		.71	
	Total insulin dose (UI)	32.5 (23.1)		29.8 (26.9)		–2.7 (18.1)		.09		38.6 (36.8)			27.2 (34.7)		–11.5 (20.6)		.09	
**Biochemical parameters, mean (SD)**																		
	Fasting plasma glucose (mg/dL)	199.4 (66.3)		130.8 (38.8)		–70.5 (72.9)		.003		231.0 (80.5)			108.4 (23.1)		–122.6 (81.5)		.004	
	HbA_1c_^b^ (%)	9.5 (1.8)		6.9 (1.2)		–2.6 (2.1)		.01		9.9 (1.7)			6.2 (1.0)		–3.7 (1.9)		.006	
	TC^c^ (mg/dL)	201.4 (57.9)		172.9 (39.9)		–26.4 (42.1)		.86		186.1 (43.4)			164.5 (31.5)		–24.7 (41.7)		.86	
	LDL-c^d^ (mg/dL)	100.1 (58.8)		91.3 (32.1)		–12.6 (60.9)		.62		94.1 (37.0)			92.1 (27.5)		–6.0 (35.3)		.62	
	HDL-c^e^ (mg/dL)	47.2 (21.4)		48.3 (11.7)		–1.6 (15.8)		.89		43.0 (19.1)			44.4 (9.8)		–0.9 (17.5)		.57	
	TG^f^ (mg/dL)	230.5 (140.6)		185.1 (114.5)		–51.7 (124.9)		.51		227.2 (161.7)			157.9 (73.1)		–73.5 (145.7)		0.51	
	eGFR^g^ (mL/min/1.72 m^2^)	95.4 (23.8)		95.3 (31.7)		4.5 (17.3)		.59		91.8 (19.6)			88.9 (18.0)		–2.5 (13.6)		.15	

^a^Difference from baseline.

^b^HbA_1c_: glycated hemoglobin.

^c^TC: total cholesterol.

^d^LDL-c: LDL cholesterol.

^e^HDL-c: HDL cholesterol.

^f^TG: triglycerides.

^g^eGFR: estimated glomerular filtration rate.

Although there was no significant change in the EsDQOL satisfaction score in the control group after 6 months of follow-up, there was a marked reduction in the EsDQOL satisfaction score in the intervention group (control: –0.7, SD 19.8 vs intervention: –13.7, SD 23.1; *P*=.02), indicating a higher satisfaction in this group after the intervention. In contrast, no relevant changes between groups were observed in the other 3 components of the EsDQOL questionnaire. However, there was a reduction of 3.8 points (from 10.6, SD 5.0 to 7.1, SD 2.7) in the intervention group in the EsDQOL diabetes-related concerns, suggesting a reduction of diabetes worries in this group. According to the ECODI scale, knowledge about diabetes increased to a higher extent in the intervention group (control: 0.3, SD 1.8 vs intervention: 1.5, SD 1.5; *P*=.001). Whereas medication adherence worsened in the control group after 6 months of follow-up, it significantly improved with the intervention (control: –8% vs intervention: 13.8%; *P*=.01). In addition, patients’ health care experiences improved in the intervention group, but not in the control group (control: –0.7, SD 1.9 vs intervention: 0.5, SD 1.4; *P*=.02; Table 4 and Multimedia Appendix 3).

Finally, no differences in the incidence of adverse events were found between groups during the follow-up (control: 5/41, 12% vs. intervention: 8/44, 18%; *P*=.44).

**Table 4:** Evolution of questionnaire scores after 6 months of follow-up by study group.

Questionnaires	Control group (n=41)					Intervention group (n=44)			
	Baseline	Study end	Difference	*P* value^a^	Baseline		Study end	Difference	*P* value^a^
EsDQOL^b^ satisfaction, mean (SD)	38.2 (12.5)	35.6 (19.4)	–0.7 (19.8)	.03	44.2 (19.2)		32.7 (18.9)	–13.7 (23.1)	.02
EsDQOL impact, mean (SD)	33.8 (10.4)	33.5 (11.8)	2.2 (11.6)	.34	31.5 (10.9)		29.5 (13.9)	–0.9 (10.3)	.65
EsDQOL social or vocational concerns, mean (SD)	11.5 (6.7)	11.8 (4.4)	1.8 (6.9)	.44	8.8 (4.8)		9.9 (4.7)	0.2 (7.0)	.11
EsDQOL diabetes-related concerns, mean (SD)	12.3 (4.5)	10.9 (4.8)	–0.9 (4.4)	.08	10.6 (5.0)		7.1 (2.7)	–3.8 (6.2)	.22
ECODI^c^, mean (SD)	8.0 (2.0)	8.4 (1.4)	0.3 (1.8)	.001	8.2 (1.7)		9.6 (1.0)	1.5 (1.5)	.001
MGL MAQ^d^ (%), mean	60	52	–8	.01	58.6		72.4	13.8	.01
IEXPAC_1-11_^e^, mean (SD)	9.0 (1.5)	8.3 (1.6)	–0.7 (1.9)	.01	8.5 (1.4)		9.3 (1.0)	0.5 (1.4)	.02
IEXPAC_12_, mean (SD)	8.5 (2.1)	8.0 (2.4)	–0.9 (2.9)	.67	6.9 (2.9)		7.6 (2.2)	0 (3.5)	.84
Questionnaire by Clarke et al [27], mean (SD)	0.5 (0.5)	—^f^	—	—	0.4 (0.5)		—	—	—

^a^Difference from baseline.

^b^EsDQOL: Spanish version of the Diabetes Quality of Life Questionnaire.

^c^ECODI: Diabetes Knowledge Scale.

^d^MGL MAQ: Morisky, Green, Levine Medication Assessment Questionnaire.

^e^IEXPAC: Instrument for Evaluation of the Experience of Chronic Patients.

^f^Only baseline data were available for this variable.

## Discussion

### Principal Findings

This multicenter, randomized, prospective study showed that, among patients with T2DM and poor metabolic control, an educational intervention through digitized systems and the use of social networks translated into greater improvements in glycemic parameters (fasting plasma glucose and HbA_1c_) and body composition and greater reductions in body weight and BMI compared with usual care (control group). In addition, satisfaction, level of knowledge about diabetes, medication adherence, and patients’ health care experiences markedly improved with this intervention.

Our study included patients treated with injectable medications, including GLP-1 receptor agonists, for T2DM. This is important, as it has been reported that more than 20% of patients with DM are taking these drugs [30]. Remarkably, in our study, both groups were well matched and comparable. Therefore, the information provided in this study is of relevance and can be easily applied to real-life patients. In fact, many studies have shown that an important proportion of patients with DM do not achieve recommended targets, indicating that new approaches are necessary to improve this situation [6,7]. This is even more necessary during periods in which medical assistance is more difficult, such as the COVID-19 pandemic. In fact, during this period, there was a worsening of the follow-up, management, and metabolic control in patients with DM [31,32].

In our study, group interventions with open training sessions through digital platforms were developed and disseminated on social networks and by patient associations. The development of new technologies is necessary in the holistic health care of patients with DM [33,34]. Digital medicine may facilitate continuous and no-burden remote monitoring of patients through the use of wearables, sensors, and smartphone technologies [35]. On the other hand, it is important to offer different communication channels that may adapt to each patient’s needs, facilitating age- and culture-specific implementation strategies, as well as content adaptations to improve efficacy and engagement among all users [36]. Thus although the main communication channels used in this study included telephone calls, WhatsApp, email, and Skype video calls, WhatsApp was the preferred application by patients to ask questions and share information with professionals, regardless of their educational and socioeconomic level, but communication through videoconferencing was particularly preferred by patients with higher educational levels compared with patients with a lower educational level or those from rural areas (data not shown). Despite the great potential of social network interventions for improving the management and prevention of complications in patients with DM, this area is just starting to develop, and more information is warranted [37]. Remarkably, in our study, although there were some differences in the educational level between groups, patients had to adequately manage social networks and apps, such as WhatsApp, Instagram, or Facebook, to be included in the study, regardless of the sociocultural level of the individuals.

Promoting healthy lifestyle changes should be considered a target for patients with DM [38]. We found that, compared with the standard of care, which includes recommendations about enhancing physical activity and healthy diet and reducing body weight in patients with obesity or overweight, the specific intervention through digitized systems and social networks reduced BMI and body weight to a higher extent. In addition, the intervention achieved greater improvements in body composition, particularly body fat mass and fat-free mass. Furthermore, the phase angle, which indicates catabolism in DM [23], was also improved by the intervention. It has been reported that defining obesity with anthropometric criteria, such as weight, BMI, or waist circumference, has little sensitivity for monitoring response to treatment, in contrast to body composition parameters [22]. Our study showed that the intervention improved both the anthropometric criteria and body composition parameters, suggesting the early benefits of this approach, even after only 6 months of follow-up.

We observed that a 6-month intervention with digitized systems and social networks was associated with a significant improvement in glycemic parameters, remarkably, with a trend toward a lower total insulin dose with less risk of hypoglycemia and greater satisfaction since rapid insulin at meals can be eliminated in many times, leading to improvements in quality of life and adherence. This indicates that better metabolic control did not depend only on optimization of antidiabetic drugs but also on a comprehensive approach. In this context, team collaboration networks, social support networks, and multidisciplinary DM care are associated with good metabolic control and should be considered in the management of these patients [39,40].

On the other hand, to actually improve the prognosis of patients with DM, it is not only necessary to attain glycemic control but also to achieve blood pressure and lipid control [41]. In our study, there were significant improvements in systolic blood pressure and lipid profile in both groups, suggesting that this intervention may provide a positive impact on the holistic approach of patients with diabetes.

It is important to ascertain not only the impact of this intervention on metabolic parameters but also the acceptance of the intervention by the patient. To fully understand this point, patients were asked to complete different questionnaires. Compared with usual care, the educational intervention through digitized systems and the use of social networks was associated with a higher satisfaction and better health care experience. Additionally, this intervention was associated with an increase in knowledge about DM, leading to a reduction in DM worries in this group. Although the mean age was around 55 years in our study and only young patients may be considered to benefit from new technologies, previous studies have also shown that they provide added value in the management even of older patients, with higher levels of satisfaction and better metabolic control [40].

Assuring good adherence to treatment is crucial for patients with chronic conditions to attain therapeutic goals. Our study showed that, whereas medication adherence worsened in the control group, it significantly improved in the intervention group. This is relevant, as this type of intervention may facilitate up-titration of medication, with fewer side effects and greater satisfaction. In fact, previous studies have shown that social networks through online communities may increase the adherence of chronic patients to the treatment prescribed by physicians [42]. In fact, it has been observed that these interventions are safe, but a multistakeholder approach is needed to reduce the digital divide [43,44]. Of note, telemonitoring of patients with T2DM has shown a reduction in health care costs as a result of better self-management of the disease by patients and greater adherence to treatment [45]. However, there are still important gaps to be resolved to ensure adequate integrated care with the help of new technologies [46]. Moreover, in the future, it would be interesting to measure the costs that interventions through telemedicine would entail for patients with T2DM in the Spanish population.

### Limitations

This study has some limitations. First, the sample size could limit the generalizability of the results. However, as this was a randomized study and the groups were well matched overall, this potential bias could be minimized. In addition, patients were recruited from the Endocrinology Departments of the Virgen Macarena Hospital (Seville) and Juan Ramón Jiménez Hospital (Huelva). Both hospitals are public university hospitals dependent on the Andalusian health service, used the same clinical protocols, and had similar medical equipment and devices. The clinical profile, socioeconomic status, or educational status of patients and health care did not differ based upon hospital site. However, in this study, patients included in the intervention group knew they were receiving an additional approach, and this could have an impact on their behavior and their answers to the questionnaires. On the other hand, to avoid bias by the physicians, data were blindly analyzed by an investigator, who was unable to know whether the data came from the control or intervention group. In addition, a longer follow-up could provide relevant information about the long-term impact of this intervention. Finally, it would be relevant to perform an economic valuation in order to quantify its efficiency in clinical practice.

### Conclusion

In conclusion, digital support and patient training in the management of diabetes lead to improved health outcomes and a better experience with the disease, translating into better quality of life and greater treatment adherence. Based on these observations, the implementation of telemedicine could highly contribute to the multidisciplinary approach to T2DM treatment. However, in the light of the limitations of this study, further studies are warranted to substantiate the effectiveness and efficiency of digital interventions in diabetes care.

## Data Availability

The data that support the findings of this study are available from the corresponding autor upon reasonable request.

## Appendix

Multimedia Appendix 1 Evolution of body weight and body mass index during the study according to the study groups.

Multimedia Appendix 2 Evolution of fasting glucose and HbA_1c_ during the study according to the study groups.

Multimedia Appendix 3 Evolution of Morisky-Green, IEXPAC and ECODI questionnaires score during the study according to the study groups.

Multimedia Appendix 4 CONSORT Checklist.

## Abbreviations

DM: diabetes mellitus
ECODI: Diabetes Knowledge Scale
eGFR: estimated glomerular filtration rate
EsDQOL: Spanish version of the Diabetes Quality of Life Questionnaire
GLP-1: glucagon-like peptide-1
HbA_1c_: glycated hemoglobin
IEXPAC: Instrument for Evaluation of the Experience of Chronic Patients
T2DM: type 2 diabetes mellitus
