# Supplementary figures and images for: Efficacy of a Digital Educational Intervention for Patients With Type 2 Diabetes Mellitus: Multicenter, Randomized, Prospective, 6-Month Follow-Up Study
Source: J Med Internet Res. 2025 Apr 10;27:e60758. doi: 10.2196/60758 (PMC12022518; doi:10.2196/60758)

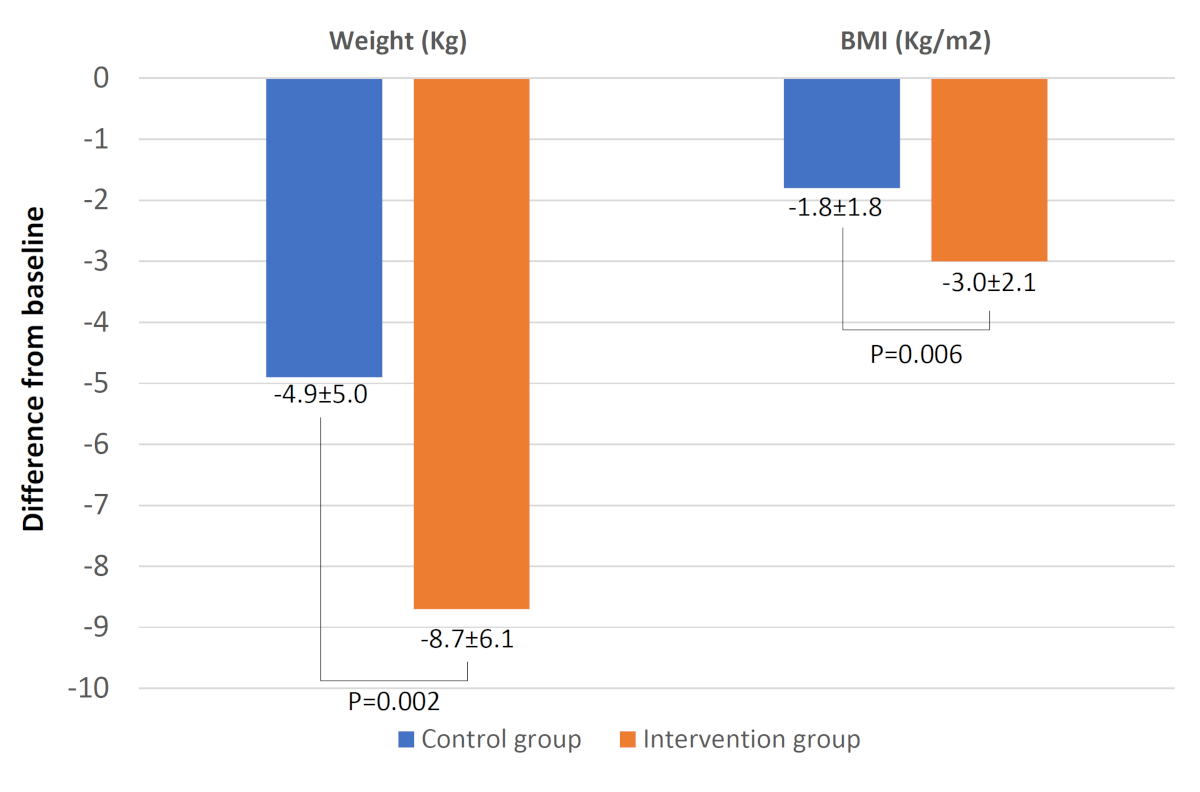

Supplement: Multimedia Appendix 1 [file jmir_v27i1e60758_app1.png]

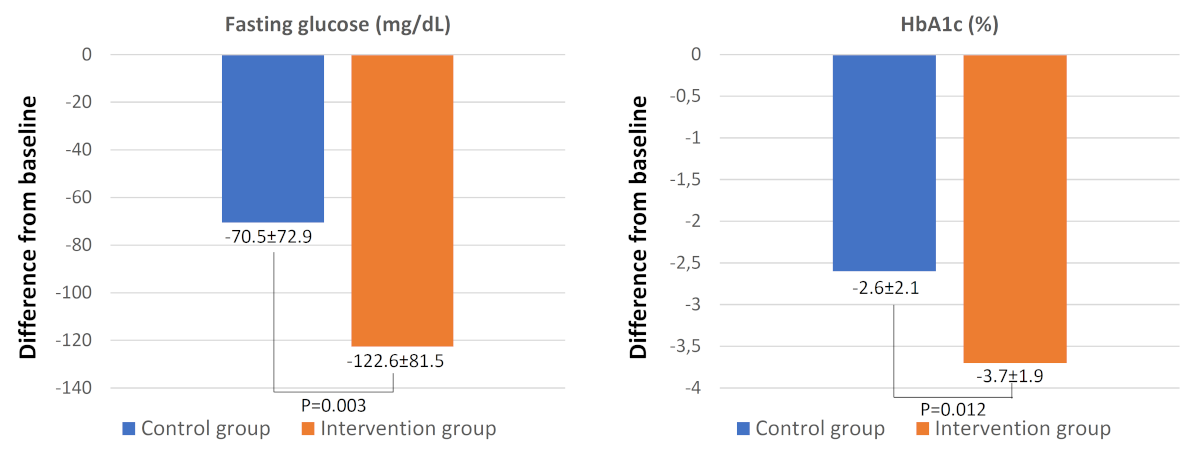

Supplement: Multimedia Appendix 2 [file jmir_v27i1e60758_app2.png]

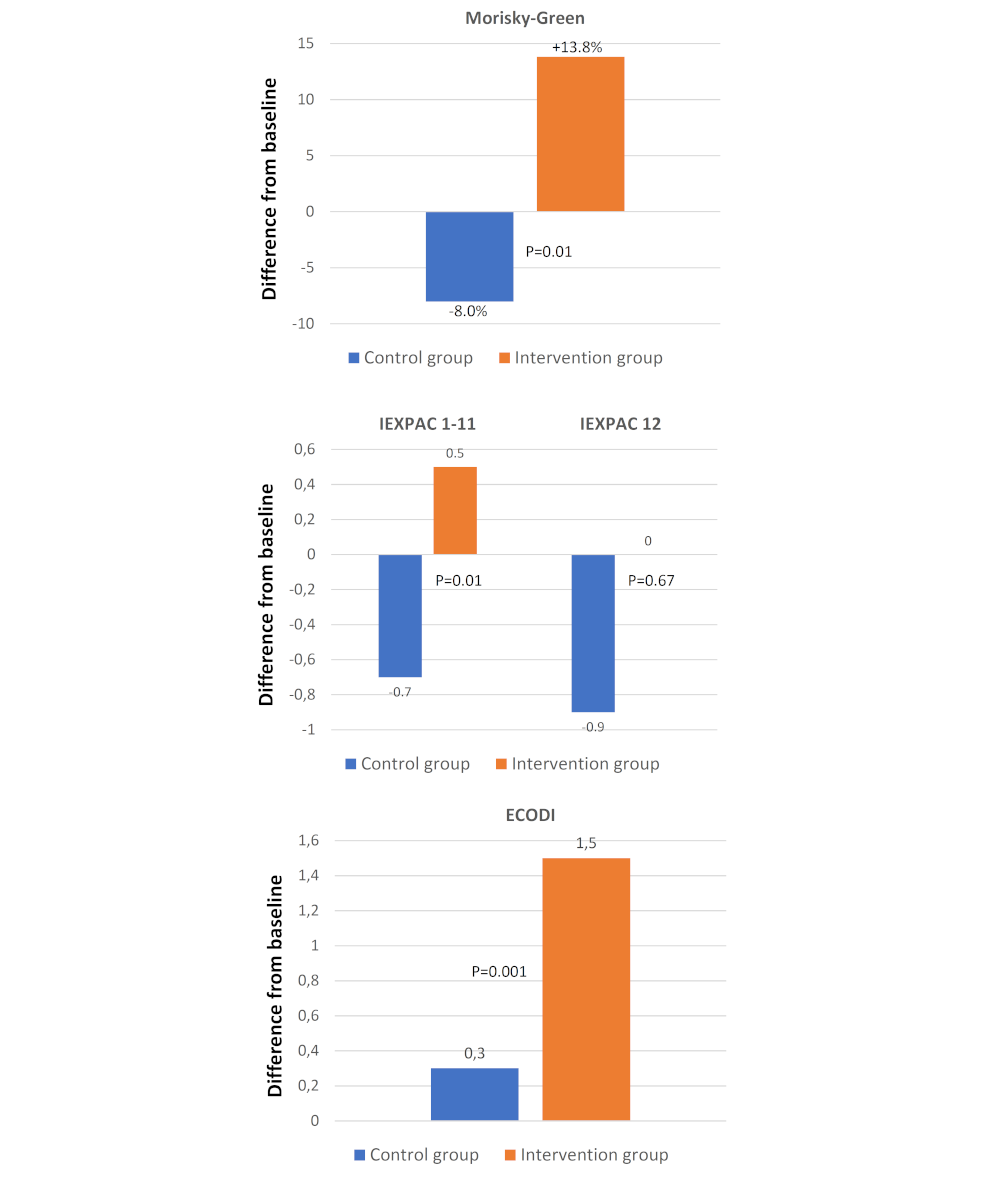

Supplement: Multimedia Appendix 3 [file jmir_v27i1e60758_app3.png]
